# Stem Cells for Cultured Meat: Cell Sources, Lineage Specification, and Biomaterial Scaffolds for Edible Tissue Engineering

**DOI:** 10.3390/ijms27146377

**Published:** 2026-07-17

**Authors:** Jihyeon Lee, Seihyun Park, Dohee Kim, Inseon Kim, Seunghun S. Lee

**Affiliations:** Department of Biomedical Engineering, Dongguk University, Seoul 04620, Republic of Korea

**Keywords:** cultured meat, muscle satellite cells, myogenic differentiation, edible scaffolds, cellular agriculture, serum-free expansion

## Abstract

Cultured meat aims to manufacture genuine animal tissue from cells in vitro, displacing the environmental and ethical liabilities of livestock slaughter. Because the final product must reproduce the fibre architecture, fat marbling, and nutrition of conventional meat, the cell—its identity, proliferative ceiling, and differentiation fidelity—is the central determinant of feasibility. This review consolidates the stem cell biology of cultured meat from a tissue engineering perspective. We first compare the principal cell sources: muscle satellite cells, which offer authentic myogenicity but limited expansion; pluripotent stem cells, which are effectively immortal but require directed differentiation; and mesenchymal, adipogenic, and fibro-adipogenic progenitors that supply fat and connective tissue. We then examine how myogenic and adipogenic commitment is controlled through growth-factor and small-molecule signalling, serum-free medium design, and co-culture strategies that recreate the multicellular composition of meat. We next survey biomaterial scaffolds—edible microcarriers, hydrogels, and decellularized plant matrices—that organize stem cells into anisotropic, perfusable, macroscale constructs, drawing on scaffold-design principles from regenerative medicine. Finally, we address bioreactor scale-up, medium cost, cell-line stability, and regulatory translation. We argue that cultured meat will advance fastest when cell source, differentiation protocol, and scaffold architecture are co-designed rather than optimized in isolation.

## 1. Introduction

The global food system faces a structural problem: feeding a growing and increasingly affluent population the protein it demands without escalating the environmental cost of doing so. Animal agriculture, and ruminant production in particular, sits at the centre of this tension. Life-cycle analyses repeatedly identify beef as among the most resource-intensive foods consumed at scale, with land use, freshwater withdrawal, and greenhouse-gas emissions per unit of edible protein far exceeding those of plant-based or novel-food alternatives [1]. Comparative assessments of “future foods” estimate that substituting animal-sourced meals with cell-based, insect-derived, or single-cell proteins could reduce global warming potential by up to 88%, land use by more than 80%, and freshwater eutrophication by more than 90%, while preserving nutritional equivalence at the meal level [1]. These figures explain why cultured meat—also termed cultivated, cell-based, or clean meat—has moved within little more than a decade from a single, widely publicized proof-of-concept hamburger to a research field with hundreds of academic groups and commercial entities, and to the first regulatory approvals for retail sale in a small number of jurisdictions [2,3].

The premise of cultured meat is conceptually simple: rather than raising and slaughtering an animal to obtain its muscle and fat, one isolates the relevant cells, expands them in a bioreactor, differentiates them into the target tissues, and assembles those tissues into a food product. In practice this is an applied form of tissue engineering, and the discipline’s foundational triad—cells, scaffolds, and signalling cues delivered within a controlled bioprocess—maps directly onto the cultured-meat workflow. Decades of accumulated knowledge in scaffold design, growth-factor delivery, and stem-cell manipulation, originally developed for regenerative medicine, can in principle be repurposed for food production [4,5]. Yet the translation is far from trivial, and the differences are instructive. A regenerative-medicine construct is implanted into a living host that supplies vascular perfusion, immune surveillance, and remodelling cues; it is produced in small numbers, at high unit cost, under clinical-grade conditions. A cultured-meat construct enjoys none of this. It must be grown to completion entirely in vitro, manufactured at commodity scale and commodity cost, rendered palatable and nutritious, and—increasingly a hard requirement—produced without animal-derived inputs anywhere in the process. These constraints reshape every design decision in the workflow.

No decision is reshaped more profoundly than the choice of cell. Meat is not a single tissue but a composite: skeletal muscle fibres, providing the bulk protein and the characteristic fibrous texture; adipose tissue, interleaved as intramuscular marbling and as discrete depots, governing flavour, juiciness, and mouthfeel; and connective tissue, the extracellular-matrix scaffold that binds the whole and contributes to bite. Each of these tissues derives from a distinct progenitor with its own biology. Consequently, the identity, expansion capacity, differentiation fidelity, and genomic stability of the starting cell population set the ceiling on what any downstream process can achieve [6,7]. A cell that cannot be expanded to sufficient numbers cannot supply a commodity product regardless of how elegant the bioreactor; a cell that will not differentiate faithfully cannot yield authentic tissue regardless of how sophisticated the scaffold.

The cell-centric framing of cultured meat has matured rapidly. The earliest demonstrations relied almost exclusively on primary muscle satellite cells harvested from livestock biopsies, prized for their authentic and efficient myogenic programme but constrained by a finite replicative lifespan that limits the number of population doublings achievable before senescence and loss of myogenicity [8,9]. The field has since broadened to embrace embryonic and induced pluripotent stem cells, mesenchymal and adipose-derived progenitors, the fibro-adipogenic progenitors resident in muscle, and a growing toolkit of engineered immortalized lines. Each occupies a different position in a four-dimensional trade-off space defined by proliferative capacity, differentiation potential, genomic and phenotypic stability, and regulatory and consumer acceptability [10,11]. In parallel, the recognition that meat is a multicellular composite has pushed the field away from muscle-only constructs and toward strategies in which several progenitor types are expanded, differentiated, and assembled together. The engineering of a self-renewing biomimetic skeletal-muscle construct from induced myogenic progenitor cells exemplifies this convergence of stem-cell reprogramming with tissue fabrication, demonstrating that the myogenic programme can be installed into a more proliferative cellular chassis and then organized into functional tissue [12]. The trajectory of the field over little more than a decade—from a single proof-of-concept patty assembled at high cost from primary bovine myoblasts, to layered fat–muscle constructs, scaled microcarrier processes, and the first retail approvals—tracks a steady shift in emphasis from “can it be done” to “can it be done at scale, affordably, and sustainably” [2,3]. That shift foregrounds a nutritional dimension that early demonstrations could afford to neglect: a credible product must not only look and taste like meat but also match its protein quality and micronutrient profile, and the cell source and differentiation protocol jointly determine whether it does. Because muscle, fat, and connective tissue each contribute distinct macro- and micronutrients, the multicellular imperative is as much a nutritional argument as a sensory one, and it reinforces the case for treating cell sourcing as a portfolio decision rather than a search for a single ideal cell [13].

**Figure 1:**
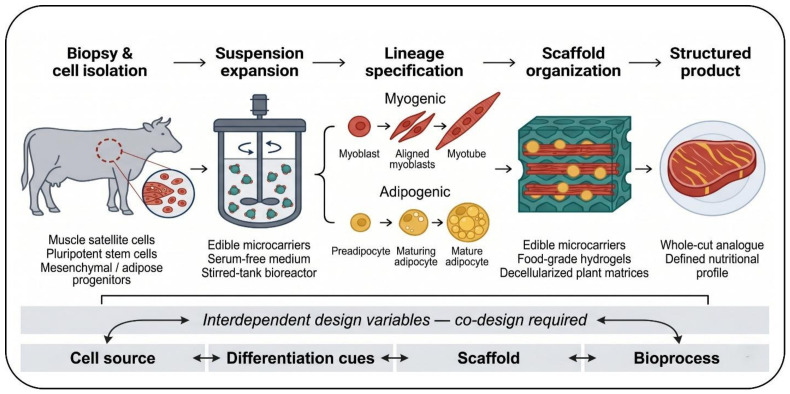
Stem or progenitor cells isolated via biopsy are expanded in serum-free media within a bioreactor. These cells undergo guided myogenic and adipogenic differentiation and are organized on edible biomaterial scaffolds to mature into structured meat. The bottom banner highlights that cell source, differentiation cues, scaffold architecture, and bioprocess configuration are interdependent variables requiring integrated co-design.

Several reviews have surveyed the cultured-meat field, addressing its cell biology comprehensively [6], its cell-sourcing workflow [7], the isolation methodology for muscle stem cells [8,11], or its bioprocess engineering [14,15]. What remains comparatively underdeveloped is an integrated account that follows the stem cell along the entire production trajectory—from source selection, through lineage specification, to its physical organization within an edible scaffold—and that evaluates each step with the design vocabulary of tissue engineering. This review is structured to fill that gap, and it adopts a deliberately cell-source-centric organization (Figure 1).

Section 2 compares the principal stem and progenitor cell sources, including the engineering of immortalized and reprogrammed lines, and frames the trade-offs that govern source selection. Section 3 examines how myogenic and adipogenic differentiation is directed, through growth-factor and small-molecule signalling, serum-free medium design, and co-culture and assembly strategies that reproduce the multicellular composition of meat. Section 4 surveys the biomaterial scaffolds—microcarriers, hydrogels, and decellularized plant matrices—that translate dispersed cells into anisotropic, perfusable, macroscale tissue, drawing explicitly on scaffold-design principles established in regenerative medicine. Section 5 addresses bioprocess scale-up, medium economics, cell-line stability, and regulatory translation. Section 6 synthesizes these threads into an outlook. Throughout, our central argument is that cell source, differentiation protocol, and scaffold architecture are not independent modules to be optimized in sequence but interdependent design variables that must be co-optimized against a defined product specification. Table 1 summarizes how the scope of the present review differs from these recent surveys.

## 2. Stem Cell Sources for Cultured Meat

The starting cell population for cultured meat must satisfy four criteria simultaneously. It must possess sufficient proliferative capacity to reach the very large cell numbers—on the order of 10^12 to 10^13 cells per production batch—that a commodity product implies. It must differentiate faithfully into the target tissue, ideally with high efficiency and synchrony. It must maintain genomic and phenotypic stability across the extended passage required to reach those numbers. And it must be obtainable through a sourcing route compatible with food regulation and consumer acceptance [7,9]. No single cell type satisfies all four criteria, and the contemporary literature is best read as a structured exploration of the resulting trade-offs (Table 2). The remainder of this section examines each major source class and the engineering interventions that attempt to relax its limitations.

### 2.1. Muscle Satellite Cells

Muscle satellite cells—the resident stem cells of adult skeletal muscle—remain the reference cell source for cultured meat because they execute the myogenic programme natively and efficiently. Anatomically, they reside in a defined niche beneath the basal lamina of the myofibre, where they are normally held in mitotic quiescence. They are identified by expression of the paired-box transcription factor Pax7, and their activation, proliferation, and differentiation are governed by the sequential deployment of the myogenic regulatory factors: Myf5 and MyoD mark commitment and proliferating myoblasts, while myogenin and MRF4 drive terminal differentiation and the fusion of mononucleated myoblasts into multinucleated myotubes that mature into contractile fibres [17,18]. Transcriptional profiling of freshly isolated satellite cells has shown that most are poised for rapid myogenic entry, with MyoD upregulation preceding the first division, even as a subpopulation retains the Pax7-high, MyoD-negative signature associated with self-renewal [18]. The quiescent state is not passive: it is actively maintained by the niche through extracellular-matrix sequestration of ligands, expression of inhibitory signalling intermediates, and post-transcriptional repression of myogenic determinants such that Myf5 transcript can be present without protein accumulation [31]. This biology has direct consequences for in vitro behaviour, because removing cells from the niche inevitably perturbs the balance between quiescence, self-renewal, and commitment.

A defining feature of satellite-cell populations is functional heterogeneity. They contain subfractions that differ in self-renewal capacity, division kinetics, and positional identity, with region-specific homeobox-gene expression contributing to anatomical patterning of myogenic potential [32]. Single-cell transcriptomic analysis of bovine muscle-derived cultures has resolved this heterogeneity directly, distinguishing genuine satellite cells from contaminating fibro-adipogenic progenitors and other muscle-resident cell types, and mapping the transitions between active, quiescent, and committed states over culture time [28]. This heterogeneity is double-edged for cultured meat: it is a resource, because the contaminating populations include the very adipogenic and matrix-producing cells a composite product needs, but it is also a liability, because undesired populations can outgrow satellite cells during prolonged proliferative culture and dilute myogenicity [28].

The practical cultured-meat literature on satellite cells has concentrated on isolation and expansion. Enzymatic dissociation of minced muscle remains the standard isolation route, but it is costly at scale and destroys extracellular-matrix components; explant-based and fully enzyme-free methods have therefore been developed that allow cells to migrate out of intact tissue fragments, better preserving cell–cell and cell–matrix interactions and yielding myogenic populations through a simplified, lower-cost process [8,33,34]. The diversity of isolation strategies—and their consequences for cell yield, purity, and downstream myogenicity—has been the subject of dedicated methodological reviews [11]. Logistically important for a distributed production model, bovine satellite cells retain their proliferative and myogenic capacity even after several days of whole-tissue cold storage, relaxing the otherwise stringent requirement that biopsies be processed immediately upon collection [19].

The dominant and well-recognized limitation of primary satellite cells is their finite replicative lifespan. Like most primary cells, they undergo a limited number of population doublings before entering replicative senescence, and—critically for cultured meat—myogenicity declines well before that ceiling is reached, as cells drift toward a non-fusing, fibroblast-like phenotype. Bioprocessing strategy must therefore be designed around this constraint rather than assuming it away, balancing the cell numbers achievable per seed train against the loss of differentiation competence [11,16]. High-throughput, label-free imaging methods now permit continuous, non-destructive quantification of both proliferation and fusion kinetics in standard well-plate formats, accelerating the empirical mapping of how isolation method, medium, substrate, and passage number jointly determine myogenic output [35]. An alternative to repeatedly harvesting native satellite cells is to generate myogenic cells de novo by reprogramming: the engineering of a self-renewing biomimetic skeletal-muscle construct from induced myogenic progenitor cells demonstrates that the myogenic programme can be installed in a chassis with greater expansion capacity, and then organized into aligned, contractile tissue [12]. Species and breed introduce a further layer of practical variation that is easy to overlook. Satellite-cell yield, proliferative kinetics, serum-free medium responsiveness, and fusion behaviour differ measurably between cattle, pigs, poultry, and fish, and even between breeds optimized for different production traits, so a protocol validated in one species rarely transfers without re-optimization [10,35]. Donor age is similarly consequential: cells from younger animals generally retain greater proliferative capacity and myogenicity, echoing the well-documented decline of satellite-cell function with age in the regenerative-biology literature [17,31]. For a distributed cultured-meat industry, these dependencies argue for early standardization of donor selection, biopsy site, and isolation protocol, because variability introduced at the cell-sourcing step propagates uncontrollably through every downstream operation.

### 2.2. Pluripotent Stem Cells

Embryonic stem cells (ESCs) and induced pluripotent stem cells (iPSCs) invert the satellite-cell trade-off. They are effectively immortal, clonally expandable, and capable in principle of generating every cell type in meat, but their myogenic and adipogenic output depends entirely on the efficiency and stability of directed differentiation protocols. Livestock pluripotent lines have historically lagged behind their murine and human counterparts, with reproducible derivation and long-term maintenance proving difficult for cattle and pigs in particular. Recent progress has been substantial: stable bovine iPSCs have been established from mesenchymal cells through reprogramming-factor cocktails supplemented with epigenetic modifiers, exhibiting prolonged self-renewal over many passages and three-germ-layer differentiation capacity [20]. Porcine pluripotent and pre-gastrulation epiblast stem cells have been stabilized under chemically defined conditions, and integration-free porcine iPSCs have been generated using episomal vectors, an important advance because transgene-free lines reduce both genomic-stability concerns and regulatory burden [21,23]. The historical promise of pluripotent sources for agriculture, long recognized in livestock biomedical models, is now being translated into cellular agriculture, where a single well-characterized, banked pluripotent line could in principle serve as the common origin for muscle, fat, and connective-tissue progenitors, simplifying quality control across the whole product [9,13,22]. The outstanding challenges are the efficiency, synchrony, and stability of myogenic specification from the pluripotent state, the cost of pluripotency-maintenance media, and the regulatory and consumer scrutiny that genomic reprogramming inevitably attracts.

### 2.3. Mesenchymal, Adipogenic, and Fibro-Adipogenic Progenitors

Because meat is not muscle alone, the progenitors of its other tissues are equally consequential. Mesenchymal stromal cells (MSCs), obtainable from bone marrow, adipose tissue, and other connective-tissue depots across livestock and avian species, are comparatively easy to isolate, expand robustly over many passages, and are multipotent—capable of myogenic, adipogenic, chondrogenic, and fibrogenic differentiation. Avian MSCs, including chicken bone-marrow- and adipose-derived populations, have been characterized specifically with cellular-agriculture applications in view, and their accessibility and multipotency make them attractive multipurpose progenitors [10,24]. Bovine adipose-derived stem cells have been expanded at litre scale as precursors of both fat and muscle tissue, demonstrating bioprocess compatibility [25]. Adipocyte progenitors deserve particular emphasis, because intramuscular and depot fat is a primary determinant of the flavour, aroma, and juiciness that distinguish meat from lean protein. The field has begun to treat adipocytes not as an afterthought but as a biomanufacturing target in their own right, “redefining” them for cultivated meat and systematically reassessing the cell-biology toolkit—progenitor selection, immortalization, induction, and lipid-loading control—available for their controlled production [26,27]. Fibro-adipogenic progenitors, the muscle-resident bipotent population long dismissed as culture contaminants, are now recognized as a legitimate and convenient endogenous source of both adipogenic cells and matrix-producing fibroblasts, and single-cell analyses have clarified how to identify and enrich them from primary muscle isolates [28]. The practical attraction of mesenchymal and adipose-derived progenitors for cultured meat is that they partially circumvent the proliferative ceiling that constrains satellite cells: many MSC populations sustain expansion over more passages before senescence, and adipose tissue in particular is an abundant, low-morbidity source that can be sampled without compromising a valuable carcass [24,25]. The trade-off is differentiation fidelity. Myogenic conversion of MSCs is generally less efficient and less synchronous than the native programme of satellite cells, often requiring forced expression of master myogenic regulators or extended induction, whereas their adipogenic conversion is robust. This asymmetry suggests a pragmatic division of labour in which satellite cells or pluripotent-derived myogenic progenitors supply the muscle compartment while MSC- and adipose-derived progenitors supply fat and connective tissue, an allocation that several multicellular assembly strategies implicitly adopt [26,27].

### 2.4. Cell-Line Engineering and Immortalization

**Figure 2:**
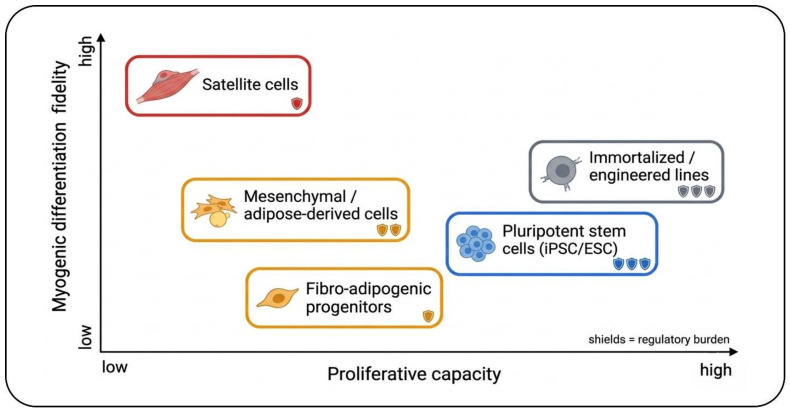
Muscle satellite cells, pluripotent stem cells, mesenchymal and adipose-derived stromal cells, fibro-adipogenic progenitors, and immortalized/engineered lines are positioned on axes of proliferative capacity and differentiation potential, with regulatory burden indicated. The general trend is a trade-off between expansion capacity and myogenic fidelity.

Spanning all of the above categories is the deliberate engineering of stable, immortalized, or otherwise enhanced cell lines, an intervention motivated directly by the proliferative ceiling of primary cells. CRISPR/Cas9-mediated knockout of the tumour-suppressor gene TP53 has yielded immortalized porcine muscle stem cells that escape senescence, exhibit markedly prolonged lifespans and elevated proliferation, and retain the ability to express muscle-specific protein markers; tellingly, the same study found that while most knockout clones remained non-tumorigenic, some acquired tumorigenic potential, illustrating in a microcosm the genomic-stability and safety concerns that immortalization raises [29]. Reprogramming and transdifferentiation offer a route to enhanced cells that does not necessarily involve permanent immortalization: transient ectopic overexpression of OCT-4 has been shown to facilitate growth-factor-induced transdifferentiation of human endothelial cells toward an osteogenic fate, a proof of principle that lineage identity can be redirected through defined, transient genetic or pharmacological intervention, and that non-myogenic cells could in principle be recruited as myogenic or adipogenic progenitor sources [36]. The integrative engineering of cultured fat similarly depends on establishing adipogenic lines with stable, consistent lipid-accumulation phenotypes across passage [30]. Taken together, these strategies make clear that cell-source selection is not a single up-front decision but an engineering programme that continues throughout the production workflow, with implications for every subsequent step [7,37]. The trade-offs across all source classes are summarized in Figure 2: in broad terms, proliferative capacity and differentiation fidelity tend to trade against one another, while every engineering intervention that relaxes one constraint tends to tighten the regulatory or genomic-stability constraint.

Building on these examples, the deliberate engineering of cell lines can be organized into four complementary strategies. First, telomerase-based immortalisation: constitutive co-expression of telomerase reverse transcriptase (TERT) with cyclin-dependent kinase 4 (CDK4) has produced immortalized bovine satellite cells that exceed 120 population doublings while retaining myogenic differentiation, furnishing a renewable, consistent starting population without disrupting tumour-suppressor pathways [38]. Second, targeted editing of muscle-growth regulators: CRISPR/Cas9 knockout of myostatin (MSTN), the negative regulator whose natural loss underlies double-muscled cattle, recapitulates muscle hypertrophy in edited livestock and is an attractive lever for raising myogenic yield in vitro [39]. Third, metabolic and trait engineering: cells can be modified to lower their dependence on costly recombinant growth factors, to improve nutrient utilization, or to bias lipid metabolism for cultured fat, shifting cost and quality burdens upstream into the cell line itself [40]. Fourth, non-genetic and spontaneous routes: prolonged culture can yield spontaneously immortalized, genetically unmodified lines, recently demonstrated for bovine fibroblasts that resolve telomere shortening through endogenous telomerase and PGC1A activation without TP53 loss, offering a non-GMO path that sidesteps much of the regulatory burden of deliberate editing [41]. Across all four, every gain in proliferative or manufacturing performance must be weighed against genomic-stability, biosafety, and regulatory-acceptance costs, a balance now framed as the central decision in cell-line development for cultivated meat [40]. Taken together, cell sourcing and its genetic enhancement define what a culture can become; the next section turns to how that latent potential is realized, by directing the chosen cells along the myogenic and adipogenic lineages that give meat its characteristic texture and flavour.

## 3. Directing Myogenic and Adipogenic Differentiation

**Figure 3:**
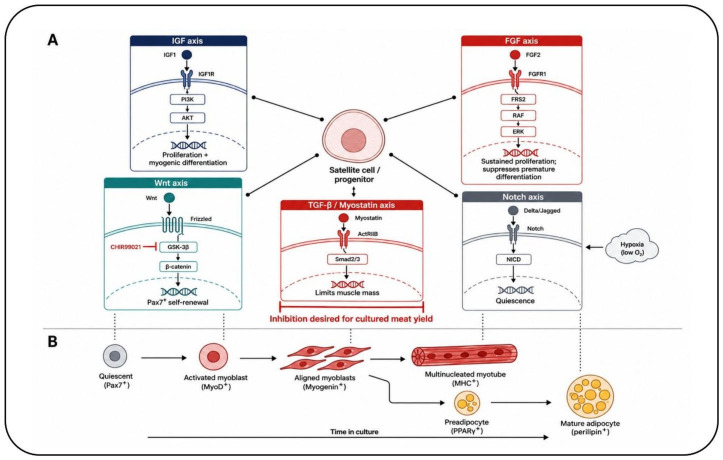
(**A**) Five key signalling axes governing the transition from progenitor proliferation to terminal differentiation: IGF1 promotes both expansion and myogenesis; FGF2 sustains expansion while suppressing premature differentiation; Wnt/β-catenin preserves self-renewal; Notch favours quiescence; and myostatin inhibition acts as a yield-enhancing lever. (**B**) Morphological trajectories of the myogenic (Pax7^+^ to MHC^+^ myotubes) and adipogenic (PPARγ^+^ to perilipin^+^ adipocytes) lineages. Dotted guides indicate the specific developmental stage at which each pathway acts.

Selecting a cell source establishes the potential of a culture; realizing that potential requires precise control of the transition from proliferation to differentiation. For cultured meat this means orchestrating at least two lineages—myogenic and adipogenic—and, for the most authentic constructs, the connective tissue that binds them, all under serum-free conditions compatible with food production and at a cost compatible with a commodity product (Figure 3).

### 3.1. Signalling Control of Myogenesis

Myogenic commitment, proliferation, and fusion are governed by an interlocking network of growth factors and hormones whose roles in satellite-cell regulation have been mapped in detail, and explicitly with cultured-meat production in mind [42]. Insulin-like growth factor 1 (IGF1) is a central anabolic node: it promotes both the proliferation and the myogenic differentiation of bovine satellite cells through the phosphatidylinositol-3-kinase/AKT axis, with overexpression studies showing dose-dependent upregulation of myosin heavy chain and myogenin and the converse on knockdown [43]. Fibroblast growth factor signalling occupies a control point that is pivotal for the entire field. FGF2 sustains satellite-cell proliferation and actively suppresses premature differentiation, holding cells in the expansion-competent state; comparative screening of commercial serum-free media has traced their differing proliferative efficacy specifically to FGF2 content acting through FGFR1 and the downstream FRS2–RAF–ERK cascade, while pharmacological FGFR1 inhibition abolishes the effect [44]. Recombinant bovine FGF1, an alternative FGF-family ligand, similarly drives proliferation and modulates mitochondrial dynamics in serum-free culture, expanding the menu of usable growth factors [45]. Wnt/β-catenin signalling tunes the balance between self-renewal and commitment: inhibition of glycogen synthase kinase-3β with the small molecule CHIR99021 expands porcine satellite cells while restraining their differentiation, preserving the Pax7-positive pool during the expansion phase, whereas downstream tankyrase inhibition produces the opposite effect [46]. Fusion itself is an actively regulated terminal step rather than a default outcome—satellite-cell-derived myoblasts synthesize netrin-4 to stimulate their own fusion through the MyoD–Myomixer axis, and manipulating such fusogenic signals offers a lever to improve myotube yield [47]. Finally, the microenvironment modulates these decisions: oxygen tension influences the self-renewal-versus-differentiation choice, with hypoxia favouring Pax7 retention and quiescence through Notch signalling, a finding with practical implications for how dissolved oxygen is controlled in expansion bioreactors [48]. Collectively, these nodes—IGF, FGF, Wnt, Notch, and the fusogenic machinery—are the levers by which the timing and efficiency of differentiation are engineered, and the small-molecule modulators among them are especially attractive because they are inexpensive, chemically defined, and free of the regulatory complications of recombinant proteins. A signalling axis of particular relevance, and one that cultured meat can exploit in a direction opposite to regenerative medicine, is the transforming growth factor-β superfamily—specifically myostatin, the endogenous negative regulator of muscle mass. Whereas clinical myology seeks to restore myostatin signalling balance, cultured-meat production benefits from suppressing it, since myostatin inhibition de-represses myoblast proliferation and hypertrophy; the naturally myostatin-deficient “double-muscled” cattle breeds are a living proof of concept, and small-molecule or genetic myostatin attenuation is an attractive lever for yield [10,42]. The non-canonical, mitogen-activated protein kinase branches of these pathways—p38 and ERK signalling—act as a switch between proliferation and differentiation, and their pharmacological modulation offers fine temporal control over when a culture is allowed to exit the cell cycle and fuse [16,47]. Because these nodes are addressable with defined small molecules rather than recombinant proteins, they are doubly attractive: they lower cost and simplify the regulatory dossier. At a mechanistic level these inputs converge on the myogenic regulatory factors through defined intermediates: active Notch signalling drives HES and HEY repressors that hold MyoD in check to enforce quiescence and self-renewal, an effect reinforced under the low-oxygen niche where hypoxia-inducible factor 1α (HIF1A) sustains Pax7 expression and restrains terminal differentiation [48,49]; canonical Wnt/β-catenin signalling, transduced through TCF/LEF, subsequently promotes the commitment-to-differentiation transition, which is why transient GSK3β inhibition expands progenitors whereas sustained pathway activity favours fusion [46]; and the p38α/β–MK2 axis acts as the terminal switch that licences cell-cycle exit and myogenin induction [16,47].

### 3.2. Serum-Free Medium Design

Fetal bovine serum is fundamentally incompatible with the ethical and economic premise of cultured meat: it is animal-derived, undefined, variable between lots, expensive, and supply-constrained, and it is consistently identified as the single largest cost driver in cultured-meat production [2]. Serum replacement is therefore a precondition for the field rather than an incremental optimization. A landmark advance was the demonstration that a low-cost serum-free medium originally developed for pluripotent stem cells (B8) could be adapted for bovine satellite cells through the addition of a single component—recombinant albumin—yielding the Beefy-9 formulation, which sustained robust expansion over at least seven passages with a doubling time near 39 h and without loss of myogenicity [50]. Subsequent work has attacked the residual cost, which is dominated by the recombinant growth factors that even serum-free media require. Conditioned-medium systems harvest growth factors secreted by inexpensive feeder cells; these have been combined with microalga-derived nutrients to create media that are simultaneously serum-free and grain-derived-nutrient-free, and further integrated with L-lactate-assimilating cyanobacteria that clear the inhibitory metabolites lactate and ammonium while replenishing glucose and pyruvate, allowing sustained myoblast proliferation without dilution [51,52]. Because comparative screening has repeatedly identified FGF2 as the decisive proliferative component, it is the rational primary target for cost reduction, whether through cheaper recombinant production, stabilized variants, or engineered autocrine supply [44,45]. Medium composition cannot be optimized in isolation from the physical substrate: attachment-promoting compounds such as vitronectin and laminin substantially improve both the proliferation rate and the purity of bovine satellite cells grown on microcarriers under serum-free conditions, and can be applied as a microcarrier coating rather than a bulk medium supplement, illustrating the tight coupling between medium and scaffold design [53].

### 3.3. Adipogenic Differentiation and Cultured Fat

Fat is not a garnish on cultured meat; intramuscular marbling and depot adipose tissue are primary determinants of flavour release, aroma generation on cooking, juiciness, and the overall sensory experience. Cultured fat has accordingly emerged as a distinct engineering target with its own literature [26,27]. The adipogenic programme is comparatively well understood—the master transcriptional regulators PPARγ and the C/EBP family drive commitment, followed by lipid-loading and maturation—and defined induction cocktails and lipid-supplementation protocols have been reassessed specifically for the requirements of cellular agriculture, where the goal is not merely to make adipocytes but to make adipocytes with a defined, food-relevant fatty-acid profile [27]. Integrative frameworks now treat adipogenic line selection, differentiation induction, and lipid-profile tuning as a single coupled problem rather than separate steps [30]. At the tissue scale, in vitro-grown adipocytes have been aggregated using food-grade binding agents into macroscale cell-cultured fat with tunable lipid composition suitable for food applications, decoupling fat production from the muscle workflow [54]. Chemical and sensory analyses of cultivated pork fat confirm that such tissue genuinely functions as a flavour enhancer when incorporated into meat alternatives, validating the premise that cultured fat has value as a stand-alone ingredient [55]. Scalable, edible production formats for fat are advancing in parallel, including edible bacterial-cellulose microcarriers coated with chitosan and plant protein that support the expansion of fat-producing cells in suspension [56]. A distinctive opportunity in cultured fat is that its composition is, in principle, a design variable rather than a fixed property of the animal. Because lipid loading can be driven with defined fatty-acid supplements, the saturated-to-unsaturated ratio and the content of nutritionally favourable species can be tuned, allowing a product that retains the sensory contribution of fat while improving its nutritional profile relative to conventional adipose tissue [30,54]. Realizing this in practice requires adipogenic lines whose lipid-accumulation phenotype is stable across passage and scale, and induction protocols that achieve mature, unilocular lipid droplets rather than the multilocular, immature morphology common in vitro [26,27]. The sensory validation of cultivated fat as a flavour enhancer confirms that the effort is worthwhile, since even modest inclusion of well-formulated cultured fat can lift the acceptability of an otherwise lean construct or a plant-protein hybrid [55,57]. Mechanistically, adipogenesis proceeds through a defined transcriptional cascade: a transient pulse of C/EBPβ and C/EBPδ induces the master regulator PPARγ, which engages a reciprocal feed-forward loop with C/EBPα to install and maintain the mature adipocyte programme, while upstream WNT and RHO-GTPase signalling gate the commitment of progenitors to the preadipocyte fate [58]. In livestock, an additional tier of Krüppel-like factors (KLF4, KLF5, and KLF15 as positive and KLF2, KLF3, and KLF7 as negative regulators) fine-tunes this cascade and intramuscular-fat deposition, identifying species-relevant control points for tuning marbling and the fatty-acid profile in cultured fat [59].

### 3.4. Co-Culture and Multicellular Assembly

Because meat is a composite tissue, the most realistic constructs integrate multiple lineages rather than perfecting one in isolation. Several assembly strategies have emerged. Multicomponent tissue has been engineered by separately culturing myogenic and adipogenic microtissues on customized scaffolds and then allowing them to adhere spontaneously into a single cohesive construct, exploiting the natural adhesive properties of the cells and their matrix [60]. A layered approach stacks aligned muscle layers with adipose layers, each formed on gelatin–soymilk scaffolds, to reproduce the laminated fat–muscle architecture characteristic of conventional cuts such as bacon or marbled steak [61]. At the manufacturing level, hybrid technologies that combine cultured cells with plant-protein matrices are converging on sensory parity by independently tuning the muscle-to-fat ratio and the connective-tissue fraction, a pragmatic route to acceptable products while pure cultured constructs mature [57]. Single-cell analyses reinforce the logic of multicellular strategies by showing that the fibro-adipogenic progenitors native to muscle can themselves supply much of the adipogenic and matrix-producing cell content these constructs require, which argues for protocols that manage and harness culture heterogeneity rather than eliminating it [28]. A frequently underappreciated component of the composite is the connective tissue and its vasculature-like channels, without which constructs cannot exceed the millimetre scale.

### 3.5. Engineering and Delivering Differentiation Cues

Beyond the identity and concentration of soluble factors, how and when cues are presented is itself an engineering variable, and here cultured meat can borrow directly from the controlled-delivery toolkit of regenerative medicine. Sequential, kinetically programmed release of multiple growth factors from a double cryogel system has been shown to enhance tissue regeneration by matching the temporal order of cue presentation to the natural differentiation timeline—proliferative cues first, differentiation and maturation cues later [62]. Heparin-functionalized cryogels sequester and present heparin-binding growth factors, including FGFs and vascular endothelial growth factor, with spatial control and protection from degradation [63]. These depot strategies are directly transferable to cultured meat: staged presentation of proliferative and then fusogenic cues from within the scaffold itself could improve myotube yield while substantially reducing the mass of expensive recombinant protein consumed per unit of product, addressing the cost problem from the delivery side rather than the supply side. Physical and biophysical cues are equally actionable. Substrate stiffness, surface topography, and mechanical loading all influence myogenic commitment and myotube alignment, and non-biochemical stimuli can be folded into differentiation protocols—static magnetic fields, for example, have been shown to promote the generation of muscle-lineage cells from both pluripotent stem cells and myoblasts, offering a reagent-free adjunct to chemical induction [64]. Directed differentiation, however, yields only a population of myotubes and adipocytes in suspension; converting that cell mass into something with the three-dimensional architecture and bite of meat is the task of the biomaterial scaffolds considered next.

## 4. Biomaterial Scaffolds for Stem Cell Organization in Cultured Meat

**Figure 4:**
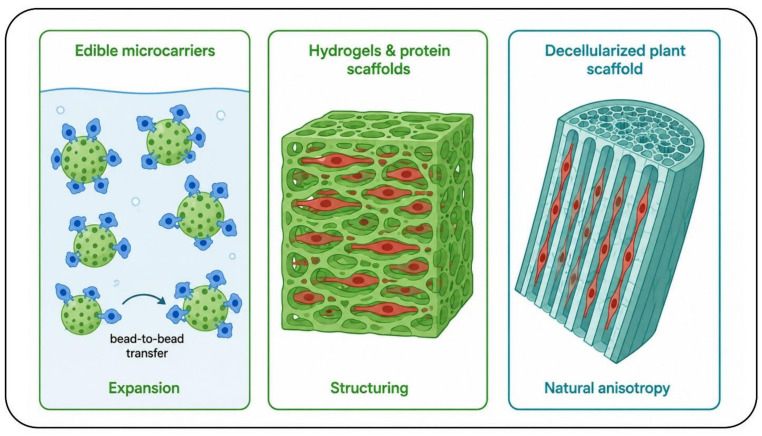
Edible microcarriers (expansion), food-grade hydrogels and protein scaffolds (structuring), and decellularized plant matrices (naturally anisotropic, perfusable scaffolds) are compared by their role in the workflow and their characteristic architecture.

Differentiated cells in suspension are not meat. Converting a population of myotubes and adipocytes into a product with the anisotropy, three-dimensionality, and bite of conventional tissue requires a scaffold: a biomaterial that organizes cells in three dimensions, presents adhesion ligands and topographical cues, permits nutrient and oxygen transport, and—a requirement unique to food—is itself edible, or else removable without compromising safety or yield [65]. Scaffold design for cultured meat draws directly on principles established over decades for bone and soft-tissue engineering: interconnected porosity to permit cell infiltration and mass transport, pore dimensions matched to the target cell, mechanical properties that approximate the native tissue modulus, controlled or arrested degradation, and surface chemistry tuned to support attachment of the chosen cell type [4]. The notion of an anatomically inspired template, in which scaffold geometry recapitulates the architecture of the native tissue it is meant to become, provides a useful framing for the whole design space [66] (Figure 4).

### 4.1. Microcarriers for Scalable Expansion

The first scaffold problem in the workflow is not structure but surface area. Muscle satellite cells, MSCs, and most other relevant cells are anchorage-dependent, and expanding them to product-relevant numbers in a stirred bioreactor requires a vast adherent surface suspended in the culture volume. Microcarriers—small beads on which cells attach and grow—provide exactly this, and they are widely regarded as the most viable route to the cell numbers a commodity product demands [67]. The cultured-meat-specific innovation is edibility. Conventional microcarriers are inedible and must be separated from the cells before consumption, adding a costly and lossy downstream step; edible microcarriers eliminate it. Emulsion-templated gelatin microparticles with independently tunable stiffness and surface topology have been demonstrated as edible microcarriers that support both the expansion and the differentiation of myogenic cells, with grooved surface textures modestly enhancing proliferation and alignment relative to smooth beads [68]. Porous gelatin microcarriers have been used to expand skeletal-muscle cells and then assembled, as cell-laden modular micro-tissues, directly into engineered meatballs without an intervening detachment step [69]. The concept of a microcarrier that simultaneously serves as a structural building block of the final tissue closely parallels the assemblable microcryogel-carrier strategy developed for site-specific regenerative medicine, in which microscale cell carriers are docked into a larger, bespoke printed scaffold [70]. Microcarrier-based processes also exploit bead-to-bead migration, in which cells transfer spontaneously to freshly added carriers without enzymatic passaging, a phenomenon that is central to the most recent process-intensification strategies and that minimizes the cell stress and reagent cost of repeated detachment [53,71].

### 4.2. Hydrogels and Edible Protein Scaffolds

Where microcarriers solve the expansion-surface problem, hydrogels address the structural problem of holding differentiated cells in a tissue-like three-dimensional environment with appropriate compliance. Skeletal muscle is a relatively soft tissue, and hydrogels can be formulated across a wide modulus range with tunable biochemistry. Gelatin-based cryogels are a particularly relevant subclass: they are macroporous, mechanically robust, sponge-like, and readily infiltrated by cells, and they have an extensive regenerative-medicine track record, including bioglass-reinforced methacrylated gelatin cryogels engineered for defect repair [72]. Because gelatin is a food-grade protein, such cryogels are natural candidates for edible muscle scaffolds, and the same fabrication chemistry transfers with minimal modification. Macroporous hybrid scaffolds that combine a load-bearing structural phase with a second phase providing sustained biochemical delivery illustrate how a single construct can supply both architecture and cue presentation, a dual function directly applicable to cultured meat [73]. The drive toward fully animal-free production has accelerated the development of plant-protein scaffolds: edible scaffolds fabricated from soy protein isolate support scalable cultured-meat production with no animal-derived components, and gelatin–soymilk composite scaffolds have been used to form the aligned muscle and adipose layers discussed in Section 3 [61,74]. The breadth of edible hydrogel and protein-scaffold chemistries now available means that scaffold selection can increasingly be matched to the specific cell source and product format rather than dictated by what is fabricable. Beyond gelatin and plant protein, the edible-hydrogel palette includes alginate, which gels rapidly under mild ionic conditions and is widely used for cell encapsulation; fibrin, which presents native cell-adhesion motifs and supports myotube formation; and polysaccharide blends that can be tuned for both mechanics and mouthfeel. Decellularized animal extracellular matrix offers the most biomimetic ligand presentation but reintroduces an animal-derived input that the field is otherwise trying to eliminate, illustrating again that scaffold choices carry regulatory and consumer-perception weight alongside their biological function [65,66]. The recurring design tension is between bioactivity and edibility: the materials that best support attachment, alignment, and differentiation are not always those with the most acceptable taste, texture, or label, and resolving that tension is a materials-science problem specific to food that has no exact counterpart in regenerative medicine [4,73].

### 4.3. Decellularized Plant Scaffolds

Among the most distinctive scaffold strategies in cultured meat is the repurposing of plant tissue. Decellularization removes the native cells from a plant while preserving its cellulose architecture, yielding a biocompatible, naturally perfusable, mechanically self-supporting, and remarkably inexpensive three-dimensional scaffold. The foundational “crossing kingdoms” demonstration showed that the vascular networks of decellularized spinach leaves and the parenchymal structures of parsley and other plants could be recellularized with mammalian endothelial, mesenchymal, and contractile cells [75,76]. For cultured meat specifically, the native anisotropy of plant tissue is a direct asset rather than an incidental feature: the three-dimensional pore structure of decellularized parsley has been shown to regulate myogenic differentiation, with fibrous, longitudinally oriented pores guiding parallel cell alignment and efficient multinucleated myotube formation, while honeycomb pore geometries connect cells in circular patterns less suited to muscle [77]. Decellularized plant scaffolds have been shown to facilitate porcine skeletal-muscle tissue engineering for cultivated-meat biomanufacturing, demonstrating the approach with a food-relevant species and cell type [78]. The generality of the platform is well supported: plant cellulose supports three-dimensional culture and lineage-specific differentiation across diverse mammalian cell types, including neural stem cells, and cellulose scaffolds derived from sources as varied as Borassus flabellifer endosperm have been validated as extracellular-matrix substitutes, often after surface functionalization or hybridization with chitosan or gelatin to improve cell attachment [79,80]. A recent advance collapses decellularization and structuring into a single step by growing muscle directly on autoclaved vegetables that have been selected and processed for biomimetic stiffness and surface micro-patterns, pointing toward minimal-processing routes to structured edible scaffolds [81].

### 4.4. Structuring, Fabrication, and Vascularization

Producing whole-cut rather than minced or comminuted product requires deliberate macroscale structuring and, inseparably, a solution to the problem of nutrient transport into thick tissue. Several advanced fabrication routes developed for regenerative medicine are directly transferable. Light-based printing of leachable salt moulds enables the facile shaping of complex porous geometries that would be difficult to achieve by casting alone, providing a route to controlled internal architecture in edible scaffolds [82]. Modular, LEGO-inspired scaffold systems allow large constructs to be assembled from standardized, individually seeded units, converting an intractable single-piece fabrication problem into a tractable assembly problem—patient-specific in the regenerative-medicine context, product-specific in the cultured-meat context [83]. The assemblable bespoke-scaffold concept, in which microscale cell carriers populate a printed macro-scaffold, offers a continuous route from microcarrier-based expansion to structured macroscale tissue without an intervening enzymatic detachment step that would otherwise stress the cells and lose yield [70]. The hardest structural barrier remains the diffusion limit: in the absence of perfusion, oxygen and nutrients penetrate only a few hundred micrometres into dense tissue, capping construct thickness and producing necrotic cores in larger pieces. Strategies for vascular-like perfusion are therefore essential to whole-cut products. Heparin-functionalized cryogels that promote neovascularization, together with progenitor cells engineered to over-express angiogenic factors such as vascular endothelial growth factor, indicate how perfusable channels and pro-angiogenic cues could be designed into thick edible constructs from the outset rather than retrofitted [63,84]. These structuring and transport considerations cannot be separated from the production-engineering questions of scale, which the next section addresses [14]. Achieving the aligned, anisotropic fibre architecture that gives meat its grain is itself a fabrication problem with several candidate solutions. Electrospun fibre mats present sub-micron topographical cues that direct myoblast elongation and parallel fusion; freeze-casting and directional ice-templating impart longitudinal pore channels to hydrogels and cryogels; and extrusion or microfluidic fibre-spinning can lay down cell-laden filaments in register. Each of these can be applied to edible polymers, and the decellularized-plant strategy can be read as nature having already solved the same anisotropy problem in cellulose [77,81]. The practical lesson is that scaffold structuring is not a single technique but a toolbox, and the right choice depends jointly on the cell source, the target product format—minced versus whole-cut—and the bioreactor in which the construct will be matured [65,66]. Scaffolds resolve the architecture of meat at laboratory scale; whether that architecture can be produced affordably, safely, and reproducibly in cubic-metre volumes is the translational question taken up in the following section.

## 5. Bioprocess Scale-Up and Translational Challenges

**Figure 5:**
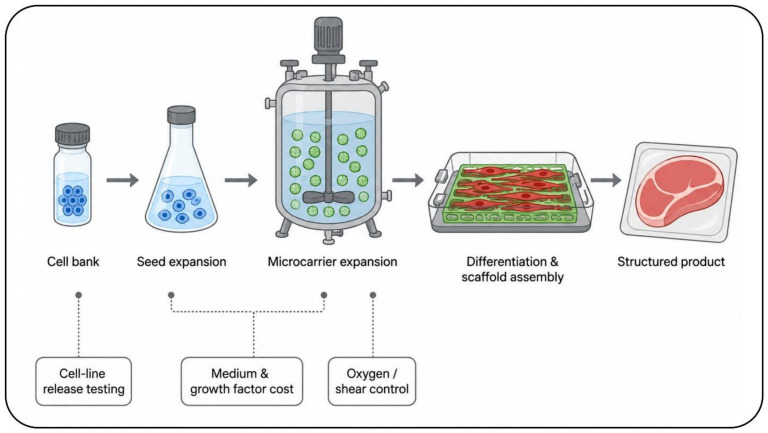
The production train runs from biopsy and cell banking, through microcarrier-based suspension expansion in a stirred-tank bioreactor, to differentiation, scaffold assembly, and the structured product, annotated with the principal cost and quality-control points (medium, growth factors, cell-line release testing).

A differentiation protocol and a scaffold design that perform at flask scale must survive translation to the cubic-metre bioreactors that a commodity product implies. Scale-up imposes its own constraints back onto cell source, medium, and scaffold, and it introduces economic, quality, and regulatory questions that ultimately gate market entry (Figure 5).

### 5.1. Bioreactors and Suspension Expansion

Reaching the trillions of cells a single production batch requires depends on moving anchorage-dependent cells out of static flasks and into controlled suspension culture. Engineering analyses of large-scale cultured-meat production consistently identify stirred-tank, microcarrier-based bioprocesses as the most credible near-term route, while highlighting the scale-dependent bottlenecks that emerge: oxygen transfer becomes limiting as the volume-to-surface ratio grows, hydrodynamic shear from impellers can damage cells and microcarriers, mixing must be sufficient for homogeneity without exceeding shear tolerance, and inhibitory metabolites accumulate unless cleared [14,15]. Concrete progress has been made on each front. Bovine adipose-derived stem cells have been expanded from spinner flasks to fully controlled litre-scale stirred-tank bioreactors with a substantial intensification fold-increase, achieved by combining bead-to-bead transfer with staged microcarrier and medium addition to expand the available surface area in situ [25]. Microcarrier-based expansion of bovine satellite cells has been intensified further by systematically optimizing the microcarrier addition strategy, the seeding density, and the confluence window for additions, with the resulting process successfully demonstrated at the multi-litre bioreactor scale [71]. These advances inherit directly from the broader bioprocessing literature on muscle stem cells, which frames the proliferative ceiling of the cells themselves as the principal engineering target that bioreactor design must work around rather than against [16,67]. Beyond the stirred tank, several alternative bioreactor configurations are under active evaluation, each with a different balance of scalability, shear, and mass transfer: packed-bed and fixed-bed reactors offer very high surface-to-volume ratios and low shear but are harder to monitor and harvest; hollow-fibre and perfusion systems support high cell densities and continuous medium exchange; and wave-mixed or vertical-wheel bioreactors reduce shear relative to impeller-driven tanks [14,15]. The differentiation phase imposes requirements distinct from expansion—often static or low-shear conditions that permit fusion, alignment, and matrix deposition—so a realistic process is likely to chain together more than one reactor type rather than relying on a single vessel. This staged view reinforces the review’s central argument, because the choice of expansion reactor, differentiation reactor, and scaffold cannot be made independently of one another [25,71].

### 5.2. Medium Economics and Sustainability

The culture medium dominates both the cost structure and the environmental footprint of cultured meat, and it is therefore the decisive variable for the field’s viability rather than a marginal one. Cradle-to-gate life-cycle assessment shows that the environmental impact of near-term cultured-meat production could actually exceed that of conventional beef if a highly refined, pharmaceutical-grade growth medium is used, because the energy and processing burden of purifying medium components is large [85]. Footprint analyses converge on the same conclusion and add an important caveat: the absence of reliable, measured process data for the proliferation and differentiation phases—and for the production of recombinant growth factors in particular—limits the accuracy of all current projections, whether optimistic or pessimistic [86]. This reframes the serum-free, conditioned-medium, and microalga- or cyanobacteria-supported media discussed in Section 3 not merely as ethical necessities but as the principal levers by which cultured meat can be made genuinely sustainable [50,51,52]. Whether cell-cultured meat from stem cells ultimately delivers on its environmental promise hinges substantially on resolving this medium problem—driving down the cost and the embodied impact of growth factors, replacing refined components with minimally processed or biologically produced alternatives, and closing nutrient loops within the bioprocess [13].

### 5.3. Cell-Line Stability and Quality Control

Extended expansion stresses genomic and phenotypic stability, and a food product manufactured from living cells inherits quality-control obligations that conventional meat does not. Immortalized lines escape the senescence ceiling but raise the questions of genetic drift across passage and, where oncogene or tumour-suppressor pathways are deliberately manipulated, of tumorigenic potential—concerns documented directly for TP53-knockout porcine muscle stem cells, some clones of which acquired tumorigenicity [29]. Even non-engineered primary cells accumulate heterogeneity and progressively lose myogenicity across passage, as discussed in Section 2, so robust process design must incorporate defined master and working cell banks, characterized passage limits, potency and identity assays, and explicit release criteria. The maturing literature on cell biology for cultured meat increasingly emphasizes precisely this quality-control dimension as a prerequisite for credible scale-up [6]. The stability of livestock pluripotent lines under chemically defined conditions is the corresponding prerequisite if such cells are to anchor production, since a single drifting or unstable founder line would compromise an entire product platform [23]. Beyond genetic change, prolonged expansion also drives progressive epigenetic drift: reproducible, passage-dependent gains and losses of DNA methylation accumulate in mesenchymal and other primary cells during culture, overlapping with the methylation changes in donor ageing and tracking the approach to replicative senescence [87,88]. Because DNA-methylation state governs lineage-specific gene expression, such drift can erode differentiation fidelity—diluting myogenic or adipogenic competence well before genetic instability is detectable—and, where it de-represses proto-oncogenic loci, may compound the transformation risk already associated with immortalized lines. Epigenetic stability should therefore be treated as an explicit release parameter for cultured-meat cell banks, monitored alongside karyotype and identity by methylation-based assays across the working passage window [88].

A food cultured from animal cells, stripped of the protection an immune system provides, is acutely vulnerable to microbial contamination, yet this dimension has attracted far less attention than cost or scale. Bacterial, fungal, viral, and mycoplasma contamination can each destroy a batch, and the nutrient-rich, antibiotic-free media that cultured meat aspires to use are an ideal substrate for adventitious organisms [89,90]. Because routine prophylactic antibiotics are incompatible with a clean-label food and risk propagating antimicrobial resistance, sterility must instead be engineered into the process through closed, aseptic bioreactor design, sterile-filtered or heat-treated media, validated cleaning- and sterilization-in-place protocols, and rapid in-line contamination monitoring [89,91]. Hazard-analysis frameworks adapted from food manufacturing—identifying critical control points across cell banking, proliferation, differentiation, and harvest—offer a structured route to managing these biological hazards, and groups have begun to map HACCP-style control points specifically onto the cultured-meat workflow [92]. Microbial control is therefore not a downstream afterthought but a design constraint that couples back to medium formulation, bioreactor configuration, and facility cost [91].

### 5.4. Regulatory Translation, Safety, and Consumer Acceptance

Technical readiness is necessary but not sufficient for cultured meat to reach a plate. Regulatory frameworks remain inconsistent across jurisdictions: a small number of markets have approved commercial sale under novel-food or equivalent pathways, while most have not yet established a clear route, and the requirements for safety data, labelling, and nomenclature differ substantially between them. Multidimensional reviews of the field emphasize that standardized safety regulation, demonstrably cost-effective production, and transparent consumer engagement together—not any one alone—determine viability [3]. Cultural and religious acceptability is a substantive rather than peripheral dimension: the Halal status of cultured meat, for instance, depends concretely on the provenance of the source cells and the composition of the culture medium, and is the subject of active scholarly and theological analysis [93]. Consumer acceptance correlates with demographic and psychological factors and, importantly, with the perceived “naturalness” of the product—a perception that the choice of cell source, the use of genetic engineering, and the nature of the scaffold can all influence, for better or worse [2,3]. Because these translational factors feed back into the earliest design decisions—favouring, for example, non-engineered cells or animal-free scaffolds even at some technical cost—they reinforce the central theme of this review: cell, cue, scaffold, and process cannot be optimized in isolation from one another or from the regulatory and social context in which the product must ultimately succeed.

The concrete regulatory landscape illustrates this unevenness. Singapore became the first jurisdiction to approve a cultured-meat product for sale in December 2020; the United States followed in June 2023, when its federal agencies cleared cultured chicken from two producers; and Israel granted the first regulatory approval of cultured beef in January 2024, with further products and jurisdictions—including an Australia–New Zealand assessment of cultured quail—advancing since [3,90]. Several governments have simultaneously moved in the opposite direction: Italy enacted a national prohibition in 2023, and individual United States such as Florida and Alabama legislated bans in 2024, while the European Union has yet to complete a novel-food authorisation and continues to apply a precautionary, EFSA-led assessment pathway [90]. This divergence places a premium on harmonized and transparent safety frameworks, standardized terminology and labelling, and shared pre-competitive safety data, which regulators across more than a dozen jurisdictions have identified as priorities for credible market entry [89].

## 6. Conclusions and Future Perspectives

Cultured meat is, at its core, a stem-cell engineering problem dressed in the constraints of food manufacturing. This review has followed the cell along the full production trajectory—from source selection, through lineage specification, to physical organization within an edible scaffold and scale-up in a bioreactor—and it arrives at a consistent conclusion: progress is gated less by any single missing technology than by the field’s tendency to optimize interdependent variables in isolation. The evidence assembled here makes the interdependence concrete. Muscle satellite cells deliver authentic, efficient myogenicity but encounter a proliferative ceiling and a parallel loss of differentiation competence; pluripotent and immortalized lines relax that ceiling but transfer the burden onto the efficiency of directed differentiation and onto genomic stability and regulatory acceptability; mesenchymal, adipose-derived, and fibro-adipogenic progenitors are not optional extras but indispensable suppliers of the fat and connective tissue that make meat taste, smell, and feel like meat [9,10]. Differentiation can now be steered with growing precision through IGF, FGF, Wnt, and Notch signalling, through serum-free media whose costs are being systematically attacked, and through staged delivery of cues from within the scaffold itself, while the multicellular composition of real tissue is increasingly reproduced through co-culture and layered assembly [30,42]. Biomaterial scaffolds—edible microcarriers, food-grade hydrogels, and decellularized plant matrices—supply the anisotropy, dimensionality, and transport that suspension culture cannot, and they do so most effectively when their architecture is matched to the chosen cell and its differentiation timeline [65,81].

**Figure 6:**
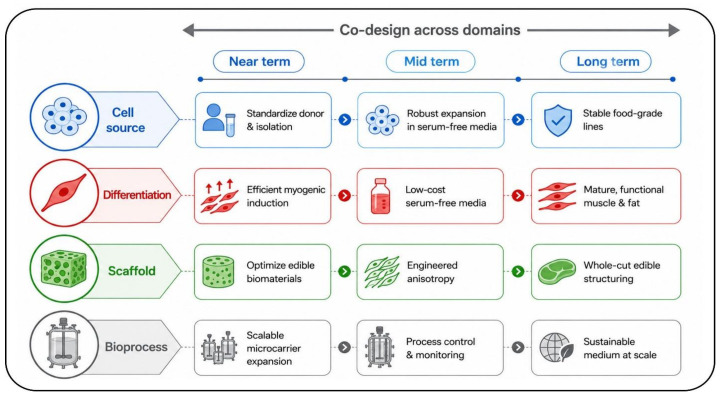
Outstanding bottlenecks and proposed near-, mid-, and long-term milestones are organized across the four interdependent design domains—cell source, differentiation, scaffold, and bioprocess—emphasizing that they must advance together.

Three priorities should define the next phase of the field. First, sustainable medium design must be solved, because life-cycle analysis shows unambiguously that it determines whether cultured meat is an environmental improvement over conventional meat at all, rather than a lateral move or a regression [85]. Second, scale-compatible structuring—the integration of microcarrier-based expansion, perfusable internal architecture, and whole-cut macroscale geometry into a single coherent process—must mature from isolated proofs of concept into robust, reproducible manufacturing, drawing deliberately on the modular and printed-scaffold strategies already developed in regenerative medicine [70,82]. Third, and most importantly, the field should commit to genuine co-design: cell source, differentiation protocol, scaffold architecture, and bioreactor configuration selected together, against an explicit product specification and an explicit regulatory and consumer context, rather than optimized sequentially and integrated as an afterthought. The convergence of stem-cell biology, biomaterials science, and three-dimensional fabrication that is already reshaping orthopedic and musculoskeletal tissue engineering provides both a template for this integrative discipline and a substantial, transferable toolkit [5,12]. If the cultured-meat field internalizes that discipline, it can move from a compelling demonstration of biological possibility toward a credible, scalable, and genuinely sustainable component of the global protein supply [6]. The principal remaining challenges and a proposed co-design roadmap are summarized in Figure 6.

To make the co-design principle operational rather than aspirational, Table 3 sets out a practical engineering framework. For each of the four design domains it lists the principal decision variables, the couplings through which a choice in one domain constrains the others, and a worked example showing how those couplings play out, converting the abstract call for co-design into a concrete checklist that can be applied against a defined product specification.

## Figures and Tables

**Table 1:** Positioning of this review relative to recent cultured-meat reviews. Coverage of each thematic dimension is graded as core (✓✓), substantial (✓), partial (◐), or not addressed (—).

Review (Ref.)	Cell Sources	Differentiation and Signalling	Scaffolds	Bioprocess Scale-Up	Regulatory and Translation	Integrative Co-Design
Martins et al. [6]	✓✓	✓	—	◐	◐	—
Reiss et al. [7]	✓✓	◐	—	◐	◐	—
Choi [8]; Lee [11]	✓ (satellite)	—	—	—	—	—
Park [14]; Pajcin [15]	◐	—	◐	✓✓	◐	—
This review	✓✓	✓✓	✓✓	✓✓	✓	✓✓

**Table 2:** Comparative summary of candidate cell sources for cultured meat.

Cell Source	Proliferative Capacity	Myogenic Fidelity	Adipogenic/Matrix Potential	Genomic and Phenotypic Stability	Sourcing and Regulatory Considerations
Muscle satellite cells	Low–moderate; finite, myogenicity lost before senescence [8,16]	High; native programme [17,18]	Low (myogenic-restricted)	Moderate; drift toward non-fusing phenotype on passage [11]	Biopsy required; non-engineered; high consumer acceptability [19]
Pluripotent stem cells (ESC/iPSC)	High; effectively unlimited [20,21]	Moderate; depends on directed differentiation [9,22]	High; multilineage [13]	Moderate; reprogramming and karyotype concerns [23]	Reprogramming raises regulatory and “naturalness” scrutiny [3]
Mesenchymal/adipose-derived stromal cells	Moderate–high [24,25]	Low–moderate; often needs forced myogenic regulators [10]	High; robust adipogenic and fibrogenic [26,27]	Moderate–high	Low-morbidity sourcing (e.g., adipose); non-engineered [24]
Fibro-adipogenic progenitors	Moderate [28]	Low	High; native adipogenic/matrix source [28]	Moderate	Co-isolated with satellite cells; manage as resource not contaminant [28]
Immortalized/engineered lines	High; senescence bypassed [29]	Moderate–high; retained in best clones [29]	Variable; line-dependent [30]	Low–moderate; drift and tumorigenicity risk [29]	Genetic modification; heaviest regulatory burden [6]

**Table 3:** For each design domain, the principal decision variables, their couplings to the other domains, and a worked example illustrate how choices propagate and must therefore be made jointly rather than sequentially.

Design Domain	Key Decision Variables	Principal Couplings to Other Domains	Worked Co-Design Example
Cell source	Source type (satellite/pluripotent/mesenchymal/FAP); immortalisation strategy; donor species, breed, and age	Sets the achievable cell number (to bioprocess), the differentiation ceiling (to differentiation), and the regulatory and consumer burden (to translation)	Adopting a TERT/CDK4-immortalized myogenic line raises population doublings but adds genomic-stability release testing and a GMO-labelling question
Differentiation and medium	Serum-free formulation; growth-factor and small-molecule schedule; target muscle-to-fat ratio	Medium cost dominates the life-cycle footprint (to bioprocess); attachment factors tie to scaffold surface chemistry; fusogenic timing sets construct maturity	Staging FGF2-driven expansion then myostatin-attenuated fusion cues delivered from the scaffold cuts the recombinant-protein mass per unit product
Scaffold	Material (edible microcarrier/hydrogel/decellularised plant); porosity and anisotropy; edibility versus bioactivity	Microcarrier surface couples to medium attachment factors; anisotropy guides differentiation alignment; thickness sets perfusion and bioreactor demands	A decellularised-plant scaffold provides edible, naturally anisotropic guidance for myotube alignment while removing a costly detachment step
Bioprocess	Reactor type (stirred/packed-bed/perfusion); microcarrier addition strategy; oxygen, shear, and metabolite control	Shear tolerance limits cell-line and microcarrier choice; differentiation needs a low-shear vessel; contamination control sets facility cost	Bead-to-bead transfer expands surface area in situ, chaining a stirred expansion reactor to a low-shear differentiation reactor without enzymatic passaging

## Data Availability

No new data were created or analyzed in this study. Data sharing is not applicable to this article.
